# Is gain in health-related quality of life after a total hip arthroplasty depended on the comorbidity burden?

**DOI:** 10.1080/17453674.2018.1457885

**Published:** 2018-04-06

**Authors:** Eva N Glassou, Alma B Pedersen, Peter K Aalund, Sebastian B Mosegaard, Torben B Hansen

**Affiliations:** 1 University Clinic for Hand, Hip and Knee Surgery, Regional Hospital West Jutland, Aarhus University, Holstebro;; 2 Department of Clinical Epidemiology, Aarhus University Hospital, Aarhus, Denmark

## Abstract

**Background and purpose** — Using patient-reported health-related quality of life (HRQoL), approximately 10% of patients report some degree of dissatisfaction after a total hip arthroplasty (THA). The preoperative comorbidity burden may play a role in predicting which patients may have limited benefit from a THA. Therefore, we examined whether gain in HRQoL measured with the EuroQol-5D (EQ-5D) at 3 and 12 months of follow-up depended on the comorbidity burden in THA patients

**Patients and methods** — 1,582 THA patients treated at the Regional Hospital West Jutland from 2008 to 2013 were included. The comorbidity burden was collected from an administrative database and assessed with the Charlson Comorbidity Index (CCI). The CCI was divided into 3 levels: no comorbidity burden, low, and high comorbidity burden. HRQoL was measured using the EQ-5D preoperatively and at 3 and 12 months’ follow-up. Association between low and high comorbidity burden compared with no comorbidity burden and gain in HRQoL was analyzed with multiple linear regression.

**Results** — All patients, regardless of comorbidity burden, gained significantly in HRQoL. A positive association between comorbidity burden and gain in HRQoL was found at 3-month follow-up for THA patients with a high comorbidity burden (coeff: 0.09 (95% CI 0.02 – 0.16)) compared with patients with no comorbidity burden.

**Interpretation** — A comorbidity burden prior to THA does not preclude a gain in HRQoL up to 1 year after THA.

Using patient-reported outcome (PRO) measures, approximately 10% of total hip arthroplasty patients (THA) report some degree of dissatisfaction after surgery (Mancuso et al. 1997, Anakwe et al. 2011, Arden et al. 2011, Rolfson et al. 2011). Dissatisfaction is primarily related to unsuccessful pain relief and fulfillment of patient expectations after the THA (Anakwe et al. 2011) and a high preoperative PRO measure indicating low impact of the underlying hip disease (Arden et al. 2011).

Several patient- and clinical-related factors have an impact on HRQoL. Age, sex, BMI, and socioeconomics all play a role (Singh and Lewallen 2009, Schafer et al. 2010, Gordon et al. 2013, Judge et al. 2013, Gordon et al. 2014, Greene et al. 2014, Mannion et al. 2015) as well as the preoperative pain and mobility (Berliner et al. 2016). Several studies have showed that HRQoL, pain, and satisfaction after a THA are affected by specific preoperative comorbidities (Singh and Lewallen 2013, Judge et al. 2013, Peter et al. 2015). However, using 3 different diagnosis-based comorbidity indices including CCI, Greene et al. (2015) found only a marginal association between a preoperative comorbidity burden and HRQoL in more than 22,000 THA patients registered in the Swedish Hip Arthroplasty Register from 2002 to 2007.

Although PROs have been increasingly used to evaluate surgery outcome from the patient perspective, this is still not part of the prospective and nationwide data collection in the Danish Hip Arthroplasty Register (Gundtoft et al. 2016). However, at the Regional Hospital of West Jutland, covering approximately 5% (285,000 inhabitants) of the Danish population, PROs have been prospectively collected on all THA patients since 2008. To our knowledge, this Danish cohort is the largest so far including PRO data following THA and thus suitable for testing the hypothesis that HRQoL depends on the comorbidity burden in a Danish setting. The purpose of this single-center study was, therefore, to examine whether the patient-reported HRQoL at 3 and 12 months was dependent on the comorbidity burden in patients treated with a THA due to osteoarthritis (OA).

## Patients and methods

### Study population and setting

Patients with a unilateral primary THA due to OA treated at the Regional Hospital West Jutland from September 2008 to December 2013 and registered in the Danish National Patient Register (DNPR) formed the basis of this study (Figure). Patients with revision or counter-lateral THA within the first year were excluded. All patients were assigned to a well-documented fast-track hip arthroplasty program (Husted et al. 2010).

**Figure:**
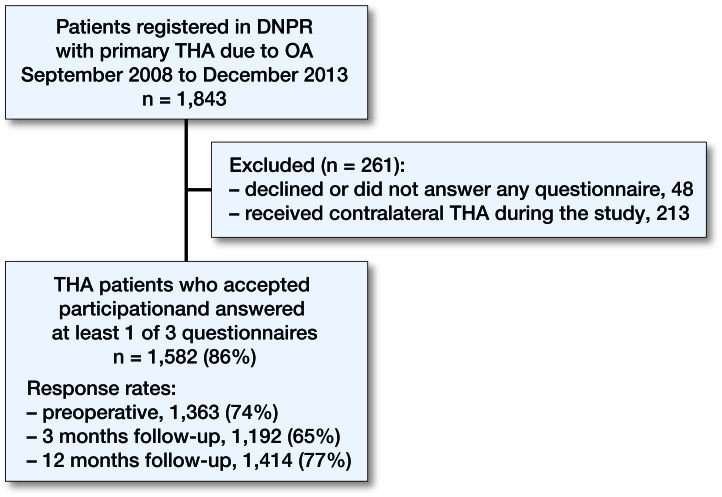
Study population. 1,582 of 1,843 patients with a primary total hip arthroplasty (THA) due to osteoarthritis (OA) treated at the Regional Hospital West Jutland from September 2008 to December 2013 and registered in the Danish National Patient Register (DNPR) were included in the study.

### Exposure

Comorbidity was established with the CCI (Charlson et al. 1987). Based on the unique 10-digit personal identification number all citizens are assigned at birth, each procedure from the cohort was linked to the DNPR to collect information about comorbidities. Each record in the DNPR holds information about hospital treatment, surgical procedures, and discharge diagnoses (Schmidt et al. 2015). All primary and secondary diagnoses from hospitalizations and outpatient visits registered as ICD-10 codes in the DNPR over a 10-year period before the primary procedure formed the basis of the CCI calculation. The CCI score was calculated by adding the points of each disease category for each procedure. All THA procedures were then divided into 3 comorbidity burden groups based on the score: patients with no comorbidity burden, patients with a low comorbidity burden (equal score 1 and 2 in the CCI), and patients with a high comorbidity burden (equal score 3 or higher in CCI). Furthermore, to see if specific diseases diverted from the CCI index score, we classified the THA procedures according to 3 specific disease groups nested in the CCI: diabetes (type I and II diabetes and diabetes with end-stage organ damage), cardiovascular diseases (myocardial infarction, congestive heart failure, peripheral vascular disease, and cerebrovascular disease) and chronic obstructive pulmonary disease (COPD).

### Outcome

The outcome was HRQoL measured with the EQ-5D 3-level version. We defined the outcome as both the EQ-5D levels and the difference between the preoperative EQ-5D score and scores at 3 and 12 months’ follow-up. EQ-5D is a short generic questionnaire, consisting of 5 dimensions (mobility, self-care, usual activities, pain/discomfort, and anxiety/depression) which can take 1 of 3 responses (no problems, some or moderate problems, and extreme problems) (http://www.euroqol.org). The responses are converted into a single weighted Danish index score with a minimum value at –0.594 and a maximum value at 1.0. The mean index score is 0.83 for 70–79-year-olds in the general Danish population (Sørensen et al. 2009). In preparation for contemporary and future research, patient-reported HRQoL outcomes including EQ-5D were collected from all THA patients at the Regional Hospital West Jutland from 2008 to 2013. Patients filled in paper questionnaires in relation to ambulatory visits preoperatively and at 3 and 12 months’ follow-up. Details in cohort recruitment have been described elsewhere (Larsen et al. 2010).

### Statistics

Patient characteristics are presented as frequencies. The EQ-5D scores are presented as means. Gain in EQ-5D was calculated as the difference between the preoperative EQ-5D score and the EQ-5D score at 3 and 12 months’ follow-up. Analysis of association between comorbidity burden and gains in EQ-5D score at 3 and 12 months’ follow-up were carried out with complete-case multiple linear regressions and adjusted for age (in 5 categorical groups: < 50, 50–59, 60–69, 70–79, and ≥80 years), sex and type of fixation (in categories: cemented THAs, uncemented THAs, and hybrid THAs). All estimates are presented with 95% confidence intervals (CI).

The gain in EQ-5D scores was tested to be normally distributed using QQ-plots. Concerning the confounders, there were only missing data in relation to type of fixation (9 observations) and we have therefore refrained from imputation of missing data. As the regression towards the mean (RTM) phenomenon may play a role when interpreting the outcome measure, we quantified the size of the RTM in relation to the 3 exposure groups according to Trochim (2006). Effect modifications from age and sex on the association between comorbidity burden and gain in EQ-5D were examined before the regression analysis. The effects were found to be homogeneous. Additionally, we tested for interaction of age and sex on the gain in EQ-5D at 3 months’ follow-up. Here we found a statistically significant but clinically irrelevant association between sex and age meaning that gain in EQ-5D was 0.003 larger per year for males than for females.

The calculation of the weighted Danish EQ-5D index scores and the analyses were performed using Stata Statistical Software, Release 12.0 (StataCorp LP, College Station, TX, USA).

### Ethics, funding, and potential conflicts of interest

Permissions were obtained from the Committee on Health Research Ethics in Central Denmark Region and from the national Danish Data Protection Agency (reference numbers: 2007-41-1197 and 2012-41-0636).No funds were received to conduct the study. The authors declare that they have no conflicts of interest.

## Results

1,582 THA patients were included (Table 1). The majority of patients (71%) had no comorbidity burden at time of surgery. 24% of the patients had a low comorbidity burden and 5% had a high comorbidity burden. In relation to non-completers (those who did not complete the questionnaires at one of the time points), there were 219 EQ-5D observations (14%) missing preoperatively. At 3 and 12 months’ follow-up, the missing EQ-5D observations accounted for 390 (25%) and 168 (11%), respectively. Due to non-completers, 1,050 and 1,227 patients, respectively, formed the basis of the analyses at 3 and 12 months of follow-up. At all 3 time points, non-completers were more often women. Preoperatively and at 3-month follow-up, non-completers were slightly older, while non-completers at 12 months of follow-up were at the same age as “completers.” Preoperatively and at 12 months of follow-up, non-completers were more often patients with a comorbidity burden. At 3 months of follow-up, the non-completers were more often patients without a comorbidity burden.

**Table 1.** Patient demography

	All THA patients		Comorbidity burden						Non-participants	
			No		Low		High			
	n = 1,582		n = 1,129		n = 379		n = 74		n = 48	
	n	%	n	%	n	%	n	%	n	%
Sex										
Female	758	48	556	49	168	44	34	46	28	58
Male	824	52	573	51	211	56	40	54	20	42
Age^a^	70	(9)	69	(9)	73	(9)	73	(8)	76	(11)
Age in categories										
10–49	49	3	46	4	3	1	0	0	2	4
50–59	186	12	145	13	35	9	6	8	4	9
60–69	563	35	442	39	103	27	18	24	12	25
70–80	583	37	385	34	160	42	38	52	15	31
80+	201	13	111	10	78	21	12	16	15	31
Year of surgery										
2008	77	5	52	5	20	5	5	7	14	29
2009	363	23	276	24	72	19	15	20	5	10
2010	278	18	206	18	59	16	13	17	4	8
2011	246	15	178	16	55	14	13	18	8	17
2012	333	21	218	19	101	27	14	19	8	17
2013	285	18	199	18	72	19	14	19	9	19
Type of fixation^b^										
Cemented THAs	216	14	131	12	67	18	18	25	19	40
Uncemented THAs	719	46	551	49	146	39	22	30	16	33
Hybrid THAs	638	40	441	39	164	43	33	45	13	27

a Age as a continuous variable, mean (SD)

b Numbers not equal to the total sum of THAs due to 9 missing observations.

### Non-participants (Table 1)

The 48 non-participants differed from the responders in relation to age, sex, comorbidity burden, and year of surgery.

### EQ-5D scores

Preoperative EQ-5D scores decreased with an increase in comorbidity burden (Table 2). At 3 months’ follow-up, the mean EQ-5D scores were approximately 0.85 irrespectively of comorbidity burden. At 12 months’ follow-up, the mean EQ-5D score for patients without a comorbidity burden reached 0.91 (SD 0.13) while the EQ-5D score had stagnated for patients with a high comorbidity burden. The gains in EQ-5D score at 3 and 12 months’ follow-up were statistically significant for all 3 comorbidity groups, but largest for patients with a high comorbidity burden. The attained gains did not, however, differ statistically significantly between the 3 comorbidity groups at either 3 or 12 months’ follow-up (3 months’ follow up: p = 0.06, 12 months’ follow up: p = 0.2). For patients with a high comorbidity burden, the gain in EQ-5D after 12 months’ follow-up decreased from 0.31 to 0.27 due to RTM. For patients with no or a low comorbidity burden, the RTM phenomenon had no effect on the gain in EQ-5D after 12 months’ follow-up.

**Table 2.** EQ-5D scores (mean (CI)) and gain (Δ) in EQ-5D between preoperative and 3 and 12 months’ follow-up in all patients and in relation to comorbidity group and disease group

Patients	Preoperative	EQ-5D scores 3 months	12 months	Δ preoperative and 3 months	Δ preoperative and 12 months
All	0.64 (0.63–0.65)	0.85 (0.64–0.86)	0.90 (0.89–0.90)	0.21 (0.20–0.22)	0.25 (0.24–0.27)
No comorbidity burden	0.65 (0.64–0.67)	0.86 (0.85–0.87)	0.91 (0.90–0.91)	0.20 (0.19–0.22)	0.25 (0.24–0.26)
Low comorbidity burden	0.61 (0.59–0.63)	0.85 (0.83–0.86)	0.87 (0.85–0.89)	0.22 (0.19–0.25)	0.25 (0.23–0.28)
High comorbidity burden	0.55 (0.49–0.61)	0.84 (0.79–0.88)	0.85 (0.81–0.89)	0.30 (0.22–0.38)	0.31 (0.23–0.38)
With diabetes	0.59 (0.53–0.64)	0.82 (0.78–0.89)	0.84 (0.80–0.88)	0.23 (0.16–0.30)	0.24 (0.18–0.30)
With cardiovascular diseases	0.58 (0.55–0.62)	0.85 (0.83–0.88)	0.86 (0.84–0.88)	0.26 (0.21–0.30)	0.27 (0.23–0.31)
With chronic obstructive pulmonary disease	0.55 (0.49–0.61)	0.81 (0.77–0.85)	0.83 (0.78–0.88)	0.25 (0.18–0.32)	0.31(0.24–0.37)

In relation to the 3 specific comorbid conditions (diabetes, cardiovascular diseases, and COPD), THA patients with COPD had the lowest preoperative EQ-5D score and postoperative score after 3 and 12 months. However, the gain in EQ-5D in COPD patients was similar to the gain achieved in patients with diabetes and cardiovascular diseases (Table 2).

### EQ-5D dimensions

For the 3 dimensions “usual activities,” pain/discomfort,” and “anxiety/depression” there was a logic association between comorbidity burden and the severity of the problems; increased comorbidity burden gave rise to increased problems with these dimensions (Table 3).

**Table 3.** Distribution of the 5 pre-surgery EQ-5D dimensions in total and according to comorbidity group

Dimensions	All THA	Comorbidity burden			p-value^a^
	patients	No	Low	High	
	n %	n %	n %	n %	
Mobility					
No problems	380 26	287 27	79 22	14 23	
Some problems	1,083 74	761 72	274 78	48 77	0.07
Extreme problems	3 < 1	3 < 1	0 –	0 –	
Self-care					
No problems	1,090 75	791 76	253 73	46 75	
Some problems	356 24	249 24	93 27	14 23	0.3
Extreme problems	7 < 1	4 < 1	2 < 1	1 2	
Usual activities					
No problems	318 22	238 23	73 21	7 11	
Some problems	997 68	772 69	232 66	43 69	< 0.001
Extreme problems	141 10	85 8	44 13	12 20	
Pain/discomfort					
No problems	29 2	21 2	7 2	1 2	
Some problems	1,112 77	833 80	240 70	39 64	< 0.001
Extreme problems	301 21	184 18	96 27	21 34	
Anxiety/depression					
No problems	1,141 79	842 82	259 75	40 67	
Some problems	281 20	177 17	85 24	19 32	< 0.001
Extreme problems	18 1	14 1	3 1	1 1	

a P-values are derived from Spearman’s rank correlation.

### Association between comorbidity burden and gain in EQ-5D

At 3 months’ follow-up, the comorbidity burden had an impact on the gain in HRQoL (CCI 1–2: coeff: 0.01 (CCI –0.02 to 0.04), CCI3+: coeff: 0.09 (CI 0.02–0.16)) compared with patients without a comorbidity burden (Table 4). After 12 months there was no difference between patients with low or high comorbidity burden compared with patients with no comorbidity burden.

**Table 4.** Associations (multiple linear regression coefficients) between comorbidity burden and gain in EQ-5D at 3 and 12 months of follow-up with 95% CI^a^

	3 months’ follow-up		12 months’ follow-up	
	Crude (CI)	Adjusted^a^ (CI)	Crude (CI)	Adjusted^a^ (CI)
No comorbidity burden	reference	reference	reference	reference
Low comorbidity burden	0.02 (–0.01 to 0.05)	0.01 (–0.02 to 0.04)	0.003 (–0.02 to 0.03)	–0.003 (–0.03 to 0.03)
High comorbidity burden	0.10 (0.03 to 0.16)	0.09 (0.02 to 0.16)	0.06 (–0.00– to 0.11)	0.05 (–0.01 to 0.11)

a Adjustments are made for age (in categories), sex, and type of fixation.

## Discussion

All THA patients regardless of comorbidity burden gained in HRQoL up to 1 year of surgery. However, patients with high comorbidity burden might gain more in HRQoL within 3 months of surgery than patients without or with low comorbidity burden.

The gain in HRQoL at 3 months’ follow-up for THA patients with a high comorbidity burden indicates that comorbidity does not unambiguously predict dissatisfaction after surgery. The stagnation in gain from 3 to 12 months of follow-up for patients with a comorbidity burden may, however, signify that the comorbid conditions matters in relation to HRQoL in the long run. This interpretation is emphasized by the lack of late gain in THA patients with 1 of the 3 specific comorbid diseases. Another relation is that the gain in HRQoL after 3 months of follow-up is primarily caused by the direct pain relief after surgery affecting all patients regardless of comorbidity burden and that the late gain from 3 to 12 months of follow-up is based on functional improvements for the benefit of patients without a comorbidity burden.

Vogl et al. (2014) concluded after examining the effect of preoperative patient characteristics in THA patients that changes in EQ-5D were mainly explained by the preoperative score: the lower the preoperative scores, the higher change in the scores. This view may also be true for our population. Despite a large gain in HRQoL for patients with a comorbidity burden compared with patients without a comorbidity burden, there was no distinct association between comorbidity burden and gain in HRQoL. Our findings are also in concordance with recent findings from the UK (Loth et al. 2017). Despite methodological limitations and a limited cohort, Loth et al. (2017) reported no between-group differences in HRQoL in 251 THA patients with and without a comorbidity burden even though both groups improved substantially in the Oxford Hip Score and the Forgotten Joint Score from pre-surgery to 12 months’ follow-up.

The Danish EQ-5D population norm is 0.83 for 70–79-year-olds (Sørensen et al. 2009). In a study examining factors influencing HRQoL after THA in Sweden and Denmark, Gordon et al. (2013) found that Danish patients had an EQ-5D score of 0.85 12 months postoperatively. In our study, we found an even higher EQ-5D score at 12 months of follow-up independent of comorbidity burden compared twith the Danish population norm and the earlier findings by Gordon et al. We explain this high self-reported HRQoL by the use of a well-defined fast-track program consisting of preoperative information with matching of expectations in relation to length of hospitalization, early supervised mobilization postoperatively, and self-rehabilitation after discharge (Larsen et al. 2008). A matching of expectations is shown to be of importance (Gandhi et al. 2009, Judge et al. 2011, Hawker et al. 2013). Surgical technique and type of both fixation and implant are shown to be associated with HRQoL (Lingard et al. 2009, Smith et al. 2012, Bagaric et al. 2014). By including type of fixation in the regression model and by restricting this study to THAs excluding hip resurfacing implants some of these issues are eliminated. Even though we have not restricted the population in relation to surgical technique, we consider the impact of these factors minimal as all patients were treated with the posterior approach.

Compared with non-comorbid patients, patients with a comorbidity burden were older. This is a potential problem because age could be a proxy for an increased comorbidity burden. But, as there were no changes in the distribution of either comorbidity groups or age groups across the study period, we interpret the findings as an unambiguous association between comorbidity burden and HRQoL and not as an association between age and HRQoL. Additionally, we included age in the regression model. We do, however, have a potential problem with the severity of the hip disease. A late stage of OA may reduce the possibility of reaching a high level of HRQoL after a THA. We did, however, find that the EQ-5D scores after 3 months of follow-up were identical across comorbidity groups, indicating a uniform disease stage. It would have been appropriate to include disease stage in the analyses, but unfortunately information on the severity of the hip disease was not available.

Our study has some limitations. The use of CCI as a measure of comorbidity may give rise to limitations. The CCI was developed to quantify the influence of comorbidity on mortality and was validated on breast cancer patients and not THA patients. Even though the index is widely used in orthopedic research it may still affect the validity. The CCI is, however, the preferred comorbidity index in Danish register research although other indices such as the Charnley classification and Elixhauser Comorbidity Index are found to be valid in relation to THA patients (Greene et al. 2015, Yurkovich et al. 2015). Another limitation in using the CCI is the omission of all psychiatric diseases except dementia. An omission of, for example, depression entails an underestimation of the found association between comorbidity and HRQoL. Additionally, the confounding may lead to differential misclassification as psychiatric diseases may affect THA patients with a high somatic comorbidity burden the most. The role of depression in relation to HRQoL after a THA is well examined in Swedish settings. Greene et al. (2016) have shown that the 10% of THA patients using antidepressants had poorer HRQoL before and after surgery and Rolfson et al. (2009) found that the preoperative anxiety/depression dimension in EQ-5D was a strong predictor for less pain relief and satisfaction 1 year after a THA.

The non-responders being different from the responders in relation to age, sex, and comorbidity is a problem. The number of non-responders is, however, limited. A more serious limitation may therefore be the missing outcome data – the non-completers. Where a plausible consequence of the non-responders being more comorbid than the responders would be in favor of the association, it is more difficult to deem the result of the non-completers. The slightly larger share of comorbid non-completers at 12 months’ follow-up could change our findings from no comorbidity impact on the gain in HRQoL to an impact at this time point. For the opposite distribution at 3 months of follow-up, where the missing HRQoL data are composed of non-comorbid healthy THA patients with no need of postoperative consultations, the association we found may be weakened. Both ways, the missing EQ-5D values are missing at random and therefore we have abstained from replacing missing HRQoL data with substituted values (Little 1992, Pedersen et al. 2017).

The prospective collection of PRO data on all THA patients at the Regional Hospital West Jutland from 2008 was far-sighted. The large population gives a unique opportunity to study HRQoL in a Danish setting which is very much needed. Considering the prospective data collection of exposure and outcome variables, the well-described fast-track program and the patient profiles, we find that the results of this single-center study can be applied in a wider Danish context.

In summary, our study demonstrates that a comorbidity burden does not preclude a gain in HRQoL after a THA. THA patients with a high comorbidity burden may after 3 months of follow-up gain the same level of HRQoL as THA patients without a comorbidity burden. Comorbid THA patients do not, however, attain the same level of HRQoL as patients without a comorbidity burden one year after the THA, but the gain in HRQoL after 3 months may still represent a vital difference for these patients in relation to self-independence, daily living, and outcome in general.

ENG, TBH, and ABP contributed to the conception of the study and the study design. ENG drafted the article. All authors contributed to the discussion and interpretation of the results. All authors revised the manuscript for intellectual content and approved the final version before submission. TBH contributed to the data collection. ENG, PKA, and SBM contributed to the data management.

*Acta* thanks Max Gordon and other anonymous reviewers for help with peer review of this study.
